# Ultrasound Morphology of Presumed Normal Anal Sacs in Dogs and Cats

**DOI:** 10.3390/ani14111684

**Published:** 2024-06-05

**Authors:** Ivana Nývltová-Pírková, Pavel Proks, Hana Moserová

**Affiliations:** Department of Diagnostic Imaging, Small Animal Clinic, Faculty of Veterinary Medicine, University of Veterinary Sciences Brno, Palackeho tr. 1946/1, 612 42 Brno, Czech Republic; nyvltovai@vfu.cz (I.N.-P.); proksp@vfu.cz (P.P.)

**Keywords:** canine, feline, anal sac, paranal sinus gland, anal gland, ultrasound, perineum

## Abstract

**Simple Summary:**

Simple Summary: Anal sacs are paired skin invaginations present in most carnivores near the lateral margins of the anus and contain combined secretions of glands located in the anal sac wall. Anal sac disease is commonly seen in small animal practices, with a higher prevalence in dogs than in cats. The diagnosis of anal sac disease is usually based on the presentation of clinical signs and physical and rectal examination. However, the clinical signs are often non-specific and may be even absent, particularly in neoplastic cases. Ultrasonographic evaluation of anal sacs in dogs and cats is a practical, readily available, non-invasive, and low-cost modality and may enable the detection of anal sac disease at an early stage, even in asymptomatic patients. This study describes a presumed normal ultrasound morphology of anal sacs in dogs and cats, as well as the feasibility, advantages, and disadvantages of ultrasound screening of anal sacs in these companion animals.

**Abstract:**

Ultrasonographic evaluation of canine and feline anal sacs is a practical promising modality to identify anal sac disease. However, limited data are available about normal ultrasound morphology of the anal sacs. This study describes the ultrasound morphology of presumed normal anal sacs in a larger sample of client-owned dogs and cats. A single-institutional prospective cross-sectional descriptive study was performed, and 137 dogs and 131 cats were included. The most common ultrasound features of the evaluated anal sacs in the dorsal plane were oval shape (99.3% of dogs and 98.5% of cats) and bilaterally similar content (94.2% of dogs and 95.4% of cats), mostly hypoechoic with diffusely hyperechoic points or unformed echogenic material (42.6% of dogs and 44% of cats). Gas in the lumen of the anal sac was detected in two dogs and mineralization in one dog. There was a statistically significant positive correlation between body weight and the size of anal sacs in dogs ≤15 kg and cats and a correlation between age and the size of anal sacs in cats. This simple method provides additional clinically significant information in detecting abnormal findings in asymptomatic patients and could contribute to the early detection of anal sac disease.

## 1. Introduction

Anal sacs, also referred to as paranal sinuses, are paired spherical skin invaginations present in most carnivores between the internal and external anal sphincter muscles on the lateral side of the anus. Anal sacs contain combined secretions of apocrine and sebaceous glands and open into the lateral margins of the anus near the anocutaneous junction through a single duct [1]. In the cat, the anal sac ducts open in a more lateral position than in the dog, and both apocrine and sebaceous glands are located in the wall of the anal sac fundus. On the contrary, only apocrine glands are found in the wall of the fundus, and sebaceous glands in the ductal wall in dogs [2,3].

The function of the anal sac is a territorial and social identification or defense [4,5], demonstrated by the described release of the anal sac secretion by frightened cats [6]. As pheromone-secreting structures, the anal sacs of carnivores have been described to be involved in chemical communication to the extent of varying the composition of the secretions during ovarian activity in bitches, making them more attractive to males [7]. The normal canine and feline anal sac content is highly variable in many parameters (color, consistency, presence or absence of solid materials) even in the same individual [8,9], without any known relation to the age, gender, and reproductive state of animals [5,10].

Anal sac disease (ASD) is common in small animal practice, with a higher prevalence in dogs than in cats [11,12]. The most common ASD is non-neoplastic (impaction, sacculitis, abscess), diagnosed in 12–15.7% of dogs [4,13,14] and in 0.4% of cats [14]. Neoplastic ASD, namely the anal sac adenocarcinoma (ASAC), accounts for approximately 2% of canine skin tumors [15,16,17]. In cats, ASAC is considered uncommon and accounts for approximately 0.5% of feline skin tumors [2].

The diagnosis of ASD is usually based on the clinical presentation and physical and rectal examination [4]. The most common clinical signs in dogs and cats are perineal irritation, swelling, and scooting [4,18]. Nevertheless, clinical signs associated with ASD are not pathognomonic, especially in anal sacculitis [4] and neoplastic diseases [16,17]. A manifestation of ASD may also be related to a change in the composition of chemical signals, potentially resulting in behavioral problems in groups of dogs [7]. In cats, the clinical signs of anal sacculitis may overlap with symptoms seen in other diseases, such as inflammatory bowel disease, and could be difficult to recognize [18]. Macroscopic examination of anal sac secretion is considered unreliable [8,9], and cytology is ineffective for diagnosing ASD in dogs [19]. In addition, the clinical manifestation of ASD can be completely absent, as evidenced by incidental findings of ASAC during rectal examination reported in 21–39% of diagnosed dogs [20,21,22].

Diagnostic imaging can aid in the early diagnosis of ASD, which is particularly important in ASAC. The size of the tumor and the presence of metastases are risk factors for decreased survival. Therefore, early diagnosis and treatment are associated with a better prognosis [21,22]. Anal sacs are not visible on plain radiographs unless they contain gas in rare cases [23]. Ultrasound is considered a practical imaging modality for the examination of anal sacs as it is inexpensive, widely available, and easy to perform given that trained staff is available [24]. Although some ultrasound features of ASD have been described in a small sample of a population of experimental dogs [25], limited data are available on the ultrasound morphology of normal canine and feline anal sacs. To the authors’ knowledge, only one study describing normal ultrasound morphology of the anal sacs on a small sample of a population of experimental presumed healthy dogs and a small sample of a population of client-owned clinically normal cats has been published [24]. On the ultrasound, the canine anal sacs appear as ellipsoidal structures lined by thin hyperechoic glandular tissue. The content of the anal sacs is generally hypoechoic with diffuse pinpoint hyperechoic foci [24,26]. In comparison, feline anal sacs are rounder with rather hyperechoic content [24].

The aim of this study is to perform ultrasound examination and describe ultrasound morphology and contents of presumed normal anal sacs in a larger sample of client-owned dogs and cats without clinical signs related to ASD.

## 2. Materials and Methods

### 2.1. Study Design

A single-institutional prospective cross-sectional descriptive study was performed. The work described in this manuscript involved the use of non-experimental client-owned animals. Clinical care for the patients followed internationally recognized high standards of veterinary clinical care. Although ethical approval from a committee was not specifically required, the study was still approved by the institutional animal care and use committee (IGA 116/2022/FVL). Informed owner consent was always obtained before the ultrasound examination.

### 2.2. Patient Selection

Client-owned dogs and cats presenting to the University of Veterinary Sciences Brno were prospectively recruited for the ultrasound examination at the Department of Diagnostic Imaging of the Small Animal Clinic between July 2018 and October 2021. The indication for ultrasound examination was a clinical presentation unrelated to ASD, and the ultrasound examination of the perianal region was included in the routine complex abdominal ultrasound examination. Other inclusion criteria for the study were the absence of anamnestic history and clinical signs possibly related to current, past, or recurrent ASD, as well as the availability of medical records, such as breed, sex, age, and weight. The dogs did not require sedation/anesthesia for the examination of the perianal region. The cats required sedation/anesthesia to render the patient cooperative. The enrolled cats were admitted for standard procedures requiring sedation/anesthesia, such as diagnostics of hip dysplasia or spaying/neutering, and ultrasonography was performed before these procedures. Sedation/anesthesia protocols were not recorded as a part of this study and were guided by individual patient needs and standard procedures used at the Small Animal Clinic.

### 2.3. Image Acquisition

**Figure 1:**
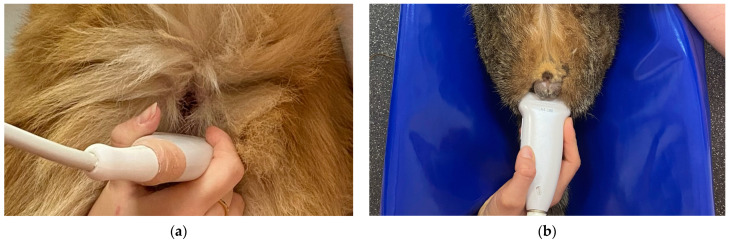
(**a**) Ultrasound examination of anal sacs in a dog. The patient is examined in a standing position with the tail reflected dorsally. (**b**) Ultrasound examination of anal sacs in a cat. The patient is examined in the dorsal recumbency in a patient positioning cradle with hind limbs pulled cranially.

**Figure 2:**
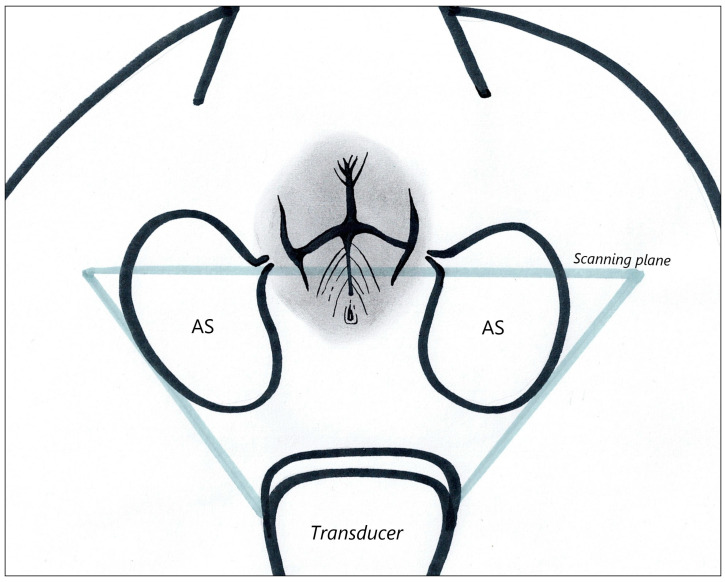
Schematic image illustrating the dorsal scanning plane used to evaluate the anal sacs in the study. AS = anal sac.

The ultrasound examination was performed by one examiner (INP) throughout the study using a Samsung RS85 (Samsung Medison Co., Ltd., Seoul, Republic of Korea) ultrasound machine with a microconvex (CF4–9 MHz) and linear transducer (LA4–18B MHz) in dogs and linear transducer in cats. The highest possible frequency was used to optimize the image quality. In dogs, the examination was performed in a standing position with the tail reflected over the dorsum (Figure 1a). Cats were positioned in dorsal recumbency in a patient positioning cradle with hind limbs pulled and fixed in the cranial direction (Figure 1b). The ultrasound examination was performed without clipping; hair was parted, and the transmission gel for the scanning was used. Dorsal and sagittal images of the anal sacs were obtained. For our research, data from the dorsal plane were used (Figure 2). All static images and short cine-loops were stored in the Picture Archiving and Communication System (PACS) of the Department of Diagnostic Imaging.

### 2.4. Image Evaluation

The ultrasound images were reviewed in DICOM viewer JiveX version 4 (Visus Health IT, Bochum, Germany) to evaluate the homogeneity, content, size, and shape of the anal sacs and the anal sac wall. In addition, unexpected abnormal (adverse) findings in the perianal region (e.g., perineal hernia, urethrolithiasis, free fluid, neoplasia of paranal sinus glands) were recorded if present.

#### 2.4.1. Content of Anal Sacs

The appearance of the left and right anal sac was subjectively evaluated in the same patient. The term “homogeneous anal sacs” has been used to describe the bilaterally similar contents of anal sacs, and the term “inhomogeneous” to describe differing contents. Subsequently, the contents of homogenous anal sacs were subjectively divided according to echogenicity into four groups: anechoic, hypoechoic, echogenic, and heteroechoic (mixed echogenicity). Other findings, such as diffuse hyperechoic points or unformed echogenic material, gas reverberations, solid material (formed non-shadowing material), and mineralization (shadowing gravity-dependent material with a hyperechoic surface), were also recorded.

#### 2.4.2. Size and Shape of Anal Sacs

The size of each anal sac was measured in the dorsal plane in cm and compared with the size of the contralateral anal sac in the same patient. For the purposes of this study, we measured the size of the anal sacs including the hypoechoic muscle layer surrounding the anal sacs’ content.

The shape of the anal sacs was evaluated based on the aspect ratio (width to height ratio) and further divided into two groups: oval and round. The round shape of the anal sac was determined as the aspect ratio equal to one. The result close to one (≤1.1) also responded to the round shape of the anal sac.

#### 2.4.3. Wall of Anal Sacs

According to a previous study, the anal sac’s tissue occasionally appears as a thin, hyperechoic line that is surrounded by the hypoechoic external sphincter muscle [24]. In our experience, the hyperechoic glandular tissue of anal sacs was visible irregularly, with the width not exceeding 1 mm. Therefore, for the purposes of this study and a more accurate measurement, the wall of the anal sac was measured together with the surrounding hypoechoic muscle. The measurement was performed in mm in the dorsal plane.

### 2.5. Statistical Analysis

All medical records and ultrasound examination data were collated in a spreadsheet using Microsoft Excel (Microsoft Excel 2019 MSO, Microsoft Corp., Redmond, WA, USA). Similar to the methodology of Colthurst et al. [27], the dogs were categorized into groups of small (≤15 kg) and medium-to-large (>15 kg) for the purposes of statistical and correlation analysis of the measured anal sac size. All analyses were performed using the statistical software IBM SPSS Statistics for Windows, version 25 (IBM Corporation, Armonk, NY, USA) or Microsoft Excel.

The normality of the datasets was assessed by a Shapiro–Wilk test and a visual inspection of histograms. In the case of normal distribution, differences in the mean values of anal sacs’ length and width, aspect ratio, and the anal sac wall width of the groups of dogs and cats were tested with the two-sample Student’s *t*-test. In the absence of normal distribution, the Mann–Whitney U-test was performed. To evaluate relationships between age and body weight of dogs and cats in study groups and the measured length and width of the anal sacs and calculated aspect ratios, correlation analysis was performed utilizing Pearson’s correlation coefficient (R) for normally distributed data and Spearman’s rank correlation coefficient (ρ) for the variables that did not follow a normal distribution. A P value of less than 0.05 was considered statistically significant. The resulting correlation was classified according to its value as very strong (0.8–1), strong (0.6–0.79), moderate (0.4–0.59), weak (0.2–0.39), and very weak (0.0–0.19) similar to the methodology of Büttelmann et al. [28].

## 3. Results

### 3.1. Study Population

The inclusion criteria met 137 dogs and 131 cats. The median age of dogs and cats at the time of enrolment was 9.1 years (range 0.3–16.2) and 0.7 years (range 0.4–14.2), respectively. The median weight was 10.5 kg (range 2–57) in dogs and 3 kg (range 1.6–6.6) in cats. There were 55 dog breeds in our study, the most common being the Jack Russel terrier (9/137) and the Dachshund (9/137). Thirty dogs were cross-breeds, which were overrepresented (30/137, 21.9%). Eighty-nine dogs were males (18 neutered), and 48 were females (26 spayed). There were four different cat breeds, and 120 cross-breeds, which were overrepresented (120/131, 91.6%). Of these, 73 were males (5 neutered), and 58 were females (1 spayed).

### 3.2. Content of Anal Sacs

**Figure 3:**
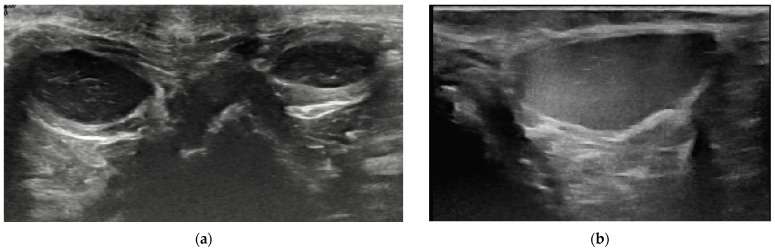
Examples of the anal sacs content and shape in dogs. (**a**) Oval-shaped anal sacs in an 18-month-old intact male cross-breed. The content is hypoechoic with hyperechoic points. Note the mild asymmetry in the size of the anal sacs. (**b**) Oval-shaped left anal sac with echogenic content in a 14-year-old spayed female Cocker spaniel.

**Figure 4:**
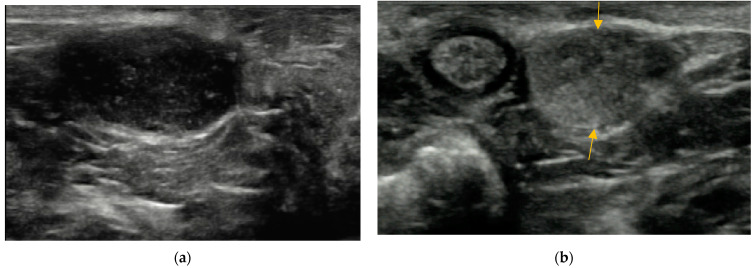
Examples of the content and shape of anal sacs in cats. (**a**) Oval-shaped right anal sac in a nine-month-old male cross-breed. The content is hypoechoic with hyperechoic points. (**b**) Round-shaped left anal sac (arrows) with echogenic content in an eight-month-old female cross-breed.

**Figure 5:**
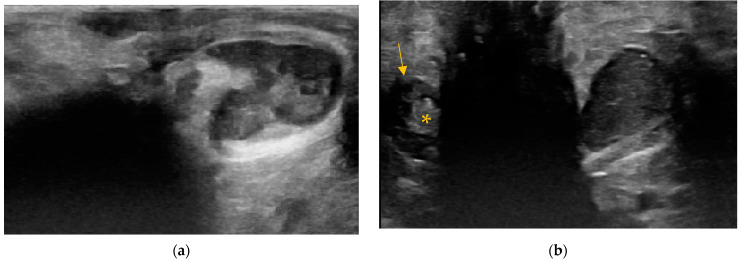
(**a**) Heteroechoic content of the anal sac in a 14-year-old female cross-breed dog. (**b**) Inhomogenous and asymmetric anal sacs in twelve-year-old male Schnauzer. The right anal sac (arrow) is small with echogenic content (asterisk), while the larger left anal sac has hypoechoic content with hyperechoic points.

The anal sacs were homogenous in 129/137 dogs (94.2%) and 125/131 cats (95.4%). The content of homogenous anal sacs was mostly hypoechoic, detected in 60/129 dogs (46.5%) and 60/125 cats (48%). Of that, hyperechoic points or unformed echogenic material was present in 55/60 dogs and 55/60 cats (42.6% and 44% of the whole study population of dogs and cats, respectively) (Figure 3a and Figure 4a). Echogenic content was detected in 49/129 (38%) dogs and 34/125 (27.2%) cats (Figure 3b and Figure 4b). Of that, hyperechoic points were present in 44/49 dogs and 27/34 cats (34.1% and 21.6% of the whole study population of dogs and cats, respectively). The anechoic content was detected in 17/129 (13.2%) dogs and 12/125 (9.6%) cats. Of that, hyperechoic points or unformed echogenic material was present in 9/17 dogs and 10/12 cats (7% and 8% of the whole study population of dogs and cats, respectively). The heteroechoic content (Figure 5a) was detected in 3/129 (2.3%) dogs and 19/125 (15.2%) cats.

Inhomogeneous anal sacs (Figure 5b) were seen in 8/137 dogs (5.8%) and 6/131 cats (4.6%), represented by different content of anal sacs (6/8 dogs, 75%; 6/6 cats, 100%) and/or the presence of unusual sediment represented by a deposit of formed hyperechoic material without acoustic shadow (2/8 dogs, 25%).

**Figure 6:**
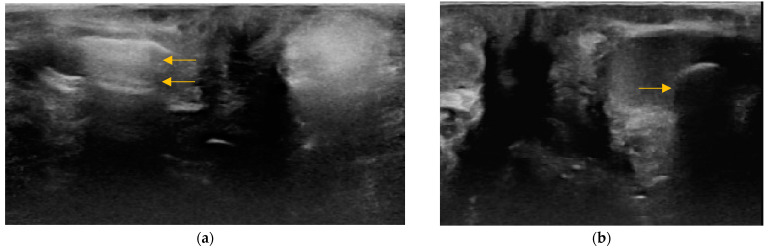
Minority findings in the anal sac content. (**a**) Presence of gas in the anal sac lumen represented by reverberations (arrows) in the gravity-non-dependent part of the anal sac in a 14-year-old female cross-breed dog. (**b**) Presence of mineralization represented by solid gravity-dependent shadowing material with hyperechoic surface (arrow) in a 14-year-old spayed female Cocker spaniel.

The presence of reverberation in the non-dependent part of anal sacs was detected in two dogs (body weight of 5 and 7.8 kg), and gravity-dependent shadowing material with the hyperechoic surface was identified in one dog (body weight of 11.6 kg) (Figure 6).

### 3.3. Size and Shape of Anal Sacs

The measured length and width of the anal sacs and the aspect ratio of the anal sacs are listed in Table 1. Based on the calculated aspect ratio, the shape of the anal sacs was marked as oval in 136/137 (99.3%) dogs and 129/131 (98.5%) cats in the dorsal plane. The rounded shape of the anal sacs was detected in one dog (0.7%) and two cats (1.5%).

Statistical analysis revealed a statistically significant difference in the mean length and width of both anal sacs between the group of cats, dogs ≤15 kg, and dogs >15 kg. No statistically significant differences were detected between left and right anal sac aspect ratios and between anal sac aspect ratios in individual study groups.

A statistically significant positive correlation between weight and length and width of both anal sacs was detected in cats and dogs ≤ 15 kg. A statistically significant correlation of age and the anal sac size was detected only in cats. The values of all significant correlation coefficients fell into the weak or very weak category. Correlations between age, body weight, and anal sacs’ size and aspect ratio are presented in Table 2.

The asymmetrical size of anal sacs was described in 16/137 (11.7%) dogs and 3/131 (2.3%) cats, different content of anal sacs in 6/137 (4.4%) dogs and 5/131 (3.8%) cats and both asymmetric size and differing contents in 2/137 (1.5%) dogs and 1/131 (0.8%) cat.

### 3.4. Wall of Anal Sacs

The measured width of the anal sacs’ wall is summarized in Table 1. The median width was 0.6 mm in cats, 1 mm in dogs ≤ 15 kg, and 1.2 (left anal sac)–1.25 (right anal sac) mm in dogs > 15 kg. There was a statistically significant difference in the mean width of the anal sac wall of both anal sacs between the group of cats, dogs ≤ 15 kg, and dogs > 15 kg. However, no statistically significant difference was detected between the right and left anal sac wall width in any of the study groups.

### 3.5. Adverse Findings

A perineal pathology was detected in 11/137 (8%) dogs. Specifically, 4 out of 11 cases (36.4%) had a perineal hernia, 4/11 (36.4%) urethrolithiasis, 1/11 (9.1%) small amount of free fluid, 1/11 (9.1%) pelvic mass, and 1/11 (9.1%) a subcutaneous nodule.

## 4. Discussion

The main aim of this study was to perform an ultrasound screening of anal sacs in a larger sample of the client-owned dog and cat population and to describe the ultrasound morphology of presumed normal anal sacs. Our results showed that the ultrasound screening of anal sacs is a simple method to obtain results with clinical importance in detecting abnormal findings in asymptomatic patients, which could contribute to the early detection of ASD.

Despite individual differences, most of the evaluated anal sacs appeared homogenous (94.2% in dogs, 95.4% in cats). The most common content of anal sacs was hypoechoic with diffuse hyperechoic points or unformed echogenic material (42.6% of dogs and 44% of cats). This finding aligns with the results of a previous study in dogs, but not in cats, where the hyperechoic content was most frequently detected [24]. In our study, the echogenic content was the second most common, with an incidence of 38% in dogs and 27.2% in cats. We assumed that this difference could be explained by the high variability of the normal content of anal sacs in dogs and cats [8,9] and the cellularity of the contents, which can affect the resulting echogenicity [24]. We also consider the age of the cats in our population to be an important factor, given that younger cats under one year of age tend to have a watery consistency of the anal sac content, according to a previous study [9]. There were 89 cats under one year of age in our study, which represents the majority (67.9%) of our study population and may have a notable influence on the overall lower echogenicity of the anal sac contents found compared to the study by Jung et al. [24].

An unusual finding in the anal sacs’ content was the presence of gas in two dogs and mineralization in one dog. The presence of gas in the anal sacs has been described on radiographs in medium-to-large dogs, with a prevalence of 6.2% in males and 4.9% in females [23]. In smaller dogs and cats, the presence of gas is assumed to be rare to none [24]. Contrary to this assumption, we found the gas-containing anal sacs in one male and one female under 15 kg (5 and 7.8 kg). Still, it is an incidental finding with unknown clinical significance [23]. The presence of mineralization in the anal sac lumen was first described on computed tomography, and the term anal sacculith was proposed to describe this finding. The prevalence in dogs is low (7.6%), with no known breed or sex predisposition and unknown clinical significance [26]. In our study, the anal sacculith was detected in the right anal sac in a 14-year-old neutered female English Cocker Spaniel with a diagnosis of transitional cell carcinoma in the bladder and urethra. This patient was treated at our clinic, and the ultrasound examination was performed after the chemotherapy.

The previously described anal sac diameter normally varies between 0.5 and 4 cm in dogs and approximately 1 cm in cats [5,29]. In the present study, the largest diameter did not exceed 2.5 cm in dogs and 1.4 cm in cats. The use of different methods to assess the size, that is, clinical examination and subjective methods in earlier studies and ultrasound in this study, could explain this discrepancy. It is also important to mention that in our assessment of the dorsal plane, the measurement was biased by the anatomical position of the anal sacs. The measured values, therefore, do not represent the actual size of the anal sacs, only the part examined in the dorsal plane. Ultrasound measurement in the sagittal plane next to the root of the tail was considered less practical due to the necessity of hair clipping and was, therefore, not performed in our study.

To the authors’ knowledge, only one study used diagnostic imaging to assess the size of anal sacs by radiography, with a major axis of 0.6–1.3 cm in cats and 0.6–1.8 cm in dogs [24]. Our study is the first to report the ultrasonographically measured size of anal sacs categorized by body weight in dogs.

The statistical analysis allowed a better understanding of the interindividual variability in anal sacs’ length and width. A correlation, albeit weak, between body weight and anal sac size was confirmed in cats and dogs ≤ 15 kg in our study. Therefore, the patient’s body weight should be considered when evaluating the size of the anal sacs on ultrasound. The correlation between weight and anal sac size was not statistically significant in the group of dogs > 15 kg. The reason may be a more significant effect of body weight in smaller patients or a greater uniformity of the group of dogs > 15 kg (selection bias). In addition, a statistically significant correlation between age and the size of the anal sacs was detected in cats.

The vast majority of examined anal sacs in our study were bilaterally symmetrical in both size and shape and homogenous in content. Asymmetry in the size of anal sacs was detected in 16 dogs and three cats, and both asymmetric size and differing contents were found in two dogs and one cat. A common explanation for anal sac distension is ASD, but the size of the anal sac can vary in healthy dogs and is not necessarily a sign of disease [4]. A previous study reached a similar conclusion, finding no correlation between the anal sac size and animals’ age or weight in both healthy and diseased animals [5]. However, in a recent study based on data reported by Dutch veterinarians, the size of anal sacs was the second most important parameter in the diagnosis of ASD after clinical signs, used in dogs and cats with 72.2% and 80% response rates, respectively [14].

Impaction of the anal sac could be a differential diagnosis for unilateral distension of the anal sac without signs of inflammation. However, the impaction is most often bilateral [4]. Impaction of the anal sac was the third most common disease diagnosed in dogs with skin diseases [12] and the most recurrent disorder of anal sac diseases [14]. In a recent study, anal sac impaction was the most common ASD in cats, with a prevalence of 0.25% [14]. A common explanation of the overall lower incidence of ASD in cats is the difference in the location of the anal sac duct opening and the differing composition of the anal sac content in dogs and cats [14,24,30,31].

In our study, the shape of the anal sacs of dogs and cats was usually oval. The resulting aspect ratio was around 1.6 in cats and 1.6–1.7 in dogs. The rounded shape of the anal sacs was detected only in one dog and two cats. This finding differs from the results of Jung et al. [24], where the shape of the anal sacs in cats is described as typically rounded. This discrepancy can be explained by the different sizes of the studied population and the use of different measurement methods. Jung et al. [24] evaluated the anal sacs on ventrodorsal and lateral radiographs after evacuation and refilling of anal sacs with contrast media was performed. We hypothesized that the overflowing of anal sacs with contrast media, as stated in the study methods, might have influenced the resulting shape. In our study, the shape was evaluated from ultrasonographic images in the dorsal plane with a naturally present amount of content. The aspect ratio was used as an objective method of shape evaluation, as opposed to the subjective shape evaluation on ultrasound images used in the above study. Similar to the echogenicity of the anal sacs’ contents, the influence of the overall young age of cats in our study population on the high prevalence of the oval shape of anal sacs in cats cannot be ruled out.

There is a lack of information about the ultrasonographic features of the anal sac wall. Jung et al. [24] described the soft tissue of the anal sac as a thin hyperechoic line surrounded by the hypoechoic external sphincter muscle. In our study, we measured the width of the anal sac wall, including the hypoechoic muscle layer, and the resulting width ranged around 0.6 mm in cats and 1–1.25 mm in dogs, with statistically significant differences in the mean width of the anal sac wall between the study groups. Measuring a structure even smaller can be imprecise and challenging not only due to the size itself but also to the occurrence of artifacts and the influence of the scanning angle on the width of structures in the image. High-resolution imaging of the anal sacs with a standard skin preparation, including hair clipping and histological confirmation of the measured size, may be necessary to accurately determine the width of the anal sac glandular tissue and differentiate it from the surrounding muscle layer.

However, this assessment is of clinical importance, especially in anal sac pathologies, where changes in the anal sac wall or surrounding soft tissue structures and associated wall thickening, increased echogenicity of the surrounding tissue, or detection of small nodules are expected. These changes could also contribute to differentiation between ASD (impaction, inflammation, abscess) with clinical importance for the following treatment, which is different for all three types of ASD [14]. This assumption is consistent with the findings of a previous study, where the anal sac wall thickening was associated with anal sacculitis in dogs [25].

The detected adverse finding was perineal pathology found in 8% of dogs, where the perineal hernia (36.4%) and urethrolithiasis (36.4%) were overrepresented. It should be noted that ASD might be the underlying cause of perineal hernia in dogs and cats [32,33]. Further studies would be necessary to evaluate the incidence of perineal hernias in dogs and cats with ASD.

This study had several limitations. The anal sacs of presumed healthy dogs and cats were evaluated in our study. Due to the absence of further diagnostics of ASD, such as cytology or histological examination, we decided not to assess the content of inhomogeneous anal sacs to eliminate the potentially diseased dogs. We are aware that the presence or absence of solid material and macroscopically different secretions between the left and right anal sac are described within the normal dog population, and they are unreliable indicators of ASD [8].

The size of the anal sacs was assessed in the dorsal plane and therefore does not represent the actual anatomical size of the anal sacs. The echogenicity of anal sacs’ content, size and shape of anal sacs, and aspect ratios in cats may be influenced by the large proportion of young cats under one year of age in our study population.

The width of the anal sac wall was measured including the hypoechoic muscle layer, due to the very small width of the anal sac tissue and possible high error rates when measuring such a thin structure. High-resolution imaging of the anal sacs with a standard skin preparation and histological confirmation of the measured size may be necessary to accurately determine the width of the anal sac glandular tissue and differentiate it from the surrounding muscle layer.

## 5. Conclusions

The present findings confirm that ultrasonography is a practical, non-invasive method that allows general assessment of the symmetry, approximate size, shape, and content of canine and feline anal sacs. The measured values in our study represent presumed normal values for ultrasonographic examination of anal sacs in the dorsal plane. The size of the canine and feline anal sacs and the width of the anal sac wall may vary depending on the body weight. Both anal sac size and content may be influenced by age in cats.

In addition, this simple method provides additional clinically significant information in detecting abnormal findings in asymptomatic patients, which may be considered a promising aspect that could contribute to the early detection of ASD.

## Figures and Tables

**Table 1:** Descriptive statistics of ultrasonographically measured parameters of anal sacs in the study population of dogs ≤ 15 kg, dogs > 15 kg, and cats. Min = minimal value; Max = maximal value; SD = standard deviation; L  =  left; R  =  right.

Dogs ≤ 15 kg (n = 86)	Mean	Median	Min	Max	SD	Confidence Interval 95%	
						Lower Limit	Upper Limit
Anal sac L length (cm)	1.27	1.30	0.70	2.00	0.28	1.21	1.33
Anal sac L width (cm)	0.78	0.75	0.40	1.50	0.22	0.73	0.83
Anal sac L wall width (mm)	1.01	1.00	0.50	1.80	0.26	0.95	1.06
Anal sac L aspect ratio	1.69	1.70	1.20	2.60	0.31	1.63	1.76
Anal sac R length (cm)	1.26	1.30	0.70	2.00	0.28	1.20	1.32
Anal sac R width (cm)	0.79	0.80	0.40	1.50	0.23	0.74	0.84
Anal sac R wall width (mm)	0.99	1.00	0.50	1.80	0.26	0.94	1.05
Anal sac R aspect ratio	1.64	1.60	1.20	2.60	0.33	1.57	1.71
**Dogs** **>** **15 kg (n = 51)**	**Mean**	**Median**	**Min**	**Max**	**SD**	**Confidence interval 95%**	
						**Lower limit**	**Upper limit**
Anal sac L length (cm)	1.65	1.60	0.90	2.50	0.36	1.54	1.75
Anal sac L width (cm)	1.03	1.10	0.40	1.70	0.29	0.95	1.11
Anal sac L wall width (mm)	1.34	1.20	0.80	2.00	0.38	1.19	1.42
Anal sac L aspect ratio	1.66	1.60	1.10	2.60	0.34	1.57	1.76
Anal sac R length (cm)	1.61	1.60	0.80	2.50	0.40	1.50	1.72
Anal sac R width (cm)	1.00	1.00	0.40	1.80	0.30	0.92	1.08
Anal sac R wall width (mm)	1.34	1.25	0.70	2.00	0.38	1.22	1.43
Anal sac R aspect ratio	1.66	1.70	1.10	2.30	0.32	1.57	1.76
**Cats (n = 131)**	**Mean**	**Median**	**Min**	**Max**	**SD**	**Confidence interval 95%**	
						**Lower limit**	**Upper limit**
Anal sac L length (cm)	0.77	0.70	0.50	1.40	0.17	0.74	0.80
Anal sac L width (cm)	0.48	0.50	0.30	0.90	0.12	0.46	0.51
Anal sac L wall width (mm)	0.61	0.60	0.40	1.10	0.15	0.59	0.64
Anal sac L aspect ratio	1.62	1.60	1.20	2.00	0.21	1.58	1.65
Anal sac R length (cm)	0.76	0.80	0.50	1.20	0.15	0.74	0.79
Anal sac R width (cm)	0.48	0.50	0.30	0.80	0.11	0.46	0.50
Anal sac R wall width (mm)	0.62	0.60	0.40	1.10	0.15	0.59	0.65
Anal sac R aspect ratio	1.60	1.60	1.00	2.00	0.22	1.56	1.64

**Table 2:** Pearson’s ^1^ and Spearman’s ^2^ correlation coefficients between body weight, age, and anal sac size and aspect ratio. A *p*-value of < 0.05 was considered statistically significant (asterisk). L = left; R = right.

Correlation Coefficients—Dogs ≤ 15 kg		Body Weight	Age
Anal sac L length	Correlation coefficient	0.341 ^1^	0.142 ^2^
	*p*-value	0.001 *	0.191
Anal sac L width	Correlation coefficient	0.223 ^2^	0.177 ^2^
	*p*-value	0.039 *	0.102
Aspect ratio L	Correlation coefficient	0.097 ^2^	−0.039 ^2^
	*p*-value	0.372	0.719
Anal sac R length	Correlation coefficient	0.266 ^1^	0.079 ^2^
	*p*-value	0.013 *	0.471
Anal sac R width	Correlation coefficient	0.227 ^2^	0.012 ^2^
	*p*-value	0.035 *	0.910
Aspect ratio R	Correlation coefficient	0.038 ^2^	0.035 ^2^
	*p*-value	0.729	0.751
**Correlation coefficients—dogs > 15 kg**		**Body weight**	**Age**
Anal sac L length	Correlation coefficient	0.308 ^2^	−0.020 ^1^
	*p*-value	0.028 *	0.887
Anal sac L width	Correlation coefficient	0.189 ^2^	0.071^1^
	*p*-value	0.184	0.620
Aspect ratio L	Correlation coefficient	0.084 ^2^	−0.139 ^2^
	*p*-value	0.556	0.331
Anal sac R length	Correlation coefficient	0.250 ^2^	−0.026 ^1^
	*p*-value	0.077	0.857
Anal sac R width	Correlation coefficient	0.164 ^2^	0.023 ^1^
	*p*-value	0.251	0.871
Aspect ratio R	Correlation coefficient	0.019 ^2^	−0.122 ^2^
	*p*-value	0.896	0.395
**Correlation coefficients—cats**		**Body weight**	**Age**
Anal sac L length	Correlation coefficient	0.240 ^2^	0.174 ^2^
	*p*-value	0.006 *	0.047 *
Anal sac L width	Correlation coefficient	0.269 ^2^	0.208 ^2^
	*p*-value	0.002 *	0.017 *
Aspect ratio L	Correlation coefficient	−0.103 ^2^	−0.092 ^2^
	*p*-value	0.243	0.298
Anal sac R length	Correlation coefficient	0.216 ^2^	0.138 ^2^
	*p*-value	0.013 *	0.117
Anal sac R width	Correlation coefficient	0.250 ^2^	0.183 ^2^
	*p*-value	0.004 *	0.036 *
Aspect ratio R	Correlation coefficient	−0.096 ^2^	−0.036 ^2^
	*p*-value	0.277	0.681

## Data Availability

The data presented in this study are stored in PACS of Small Animal Clinic, University of Veterinary Sciences Brno. The data are not publicly available due to privacy and security protection. An Excel spreadsheet is available from the authors upon request.
